# Multivariate competing endogenous RNA network characterization for cancer microRNA biomarker discovery: a novel bioinformatics model with application to prostate cancer metastasis

**DOI:** 10.1093/pcmedi/pbac001

**Published:** 2022-01-10

**Authors:** Yuxin Lin, Xin Qi, Jing Chen, Bairong Shen

**Affiliations:** Institutes for Systems Genetics, Frontiers Science Center for Disease-Related Molecular Network, West China Hospital, Sichuan University, Chengdu 610212, China; Department of Urology, the First Affiliated Hospital of Soochow University, Suzhou 215000, China; Center for Systems Biology, Soochow University, Suzhou 215006, China; School of Chemistry and Life Sciences, Suzhou University of Science and Technology, Suzhou 215011, China; Center for Systems Biology, Soochow University, Suzhou 215006, China; Institutes for Systems Genetics, Frontiers Science Center for Disease-Related Molecular Network, West China Hospital, Sichuan University, Chengdu 610212, China

**Keywords:** miRNA biomarker, competing endogenous RNA, network characterization, single-line regulation, prostate cancer metastasis

## Abstract

**Background:**

MicroRNAs (miRNAs) are post-transcriptional regulators with potential as biomarkers for cancer management. Data-driven competing endogenous RNA (ceRNA) network modeling is an effective way to decipher the complex interplay between miRNAs and spongers. However, there are currently no general rules for ceRNA network-based biomarker prioritization.

**Methods and results:**

In this study, a novel bioinformatics model was developed by integrating gene expression with multivariate miRNA-target data for ceRNA network-based biomarker discovery. Compared with traditional methods, the structural vulnerability in the human long non-coding RNA (lncRNA)–miRNA–messenger RNAs (mRNA) network was comprehensively analyzed, and the single-line regulatory or competing mode among miRNAs, lncRNAs, and mRNAs was characterized and quantified as statistical evidence for miRNA biomarker identification. The application of this model to prostate cancer (PCa) metastasis identified a total of 12 miRNAs as putative biomarkers from the metastatic PCa-specific lncRNA–miRNA–mRNA network and nine of them have been previously reported as biomarkers for PCa metastasis. The receiver operating characteristic curve and cell line qRT-PCR experiments demonstrated the power of *miR-26b-5p, miR-130a-3p*, and *miR-363-3p* as novel candidates for predicting PCa metastasis. Moreover, PCa-associated pathways such as prostate cancer signaling, *ERK/MAPK* signaling, and *TGF-β* signaling were significantly enriched by targets of identified miRNAs, indicating the underlying mechanisms of miRNAs in PCa carcinogenesis.

**Conclusions:**

A novel ceRNA-based bioinformatics model was proposed and applied to screen candidate miRNA biomarkers for PCa metastasis. Functional validations using human samples and clinical data will be performed for future translational studies on the identified miRNAs.

## Introduction

The development of high-throughput sequencing and translational informatics offers a new frontier in integrating multi-omics data for computer-aided biomarker discovery.^1^, ^2^ It is widely acknowledged that cancer is a multiple-gene disease, and the interplay between genetic components contributes to the diversity and complexity of cancer phenotypes. Hence deciphering multivariate regulatory patterns in a network manner promotes systematic understanding of cancer genesis and progression.

MicroRNAs (miRNAs) are known to be a class of small non-coding RNAs with the potential to regulate gene expression at the post-transcriptional level. Accumulating studies indicated that long non-coding RNAs (lncRNAs) could act as competing endogenous RNAs (ceRNAs) to regulate miRNA targets through competing interactions with messenger RNAs (mRNAs).^3^ Based on the construction and characterization of miRNA-mediated ceRNA networks, functional genes and modules could be computationally identified and validated for cancer application.^4^ For example, Wang *et al*. integrated miRNA-target relationships and cancer expression profiles to quantify the crosstalk among miRNAs, lncRNAs, and mRNAs in the lncRNA–miRNA–mRNA network. They defined a competitive activity score and extracted lncRNA–miRNA–mRNA triplets associated with global patterns of cancers as the prognostic biomarker.^5^ Liu *et al*. compared ceRNA networks for different ages of women with breast cancer and found prognostic biomarkers specific to each age group.^6^ Li *et al*. extracted survival-related lncRNA–miRNA–mRNA networks for immune infiltration in colorectal cancer by evaluating the expression and prognostic power of dysregulated miRNAs for model training.^7^Zhang *et al*. constructed a p53-mediated ceRNA network and identified several master miRNAs regulating p53 signaling and the mechanism of hepatocellular carcinoma therapeutics.^8^ Kang *et* al. analyzed ceRNA networks specific to diffuse large B-cell lymphoma and Hodgkin's lymphoma to screen pivotal lncRNAs for understanding the heterogeneity between lymphoma subtypes.^9^ In terms of prostate cancer (PCa), key players and mechanisms in PCa occurrence and immune infiltrates were screened via integrated gene expression and ceRNA network analyses.^10^, ^11^

**Figure 1.**
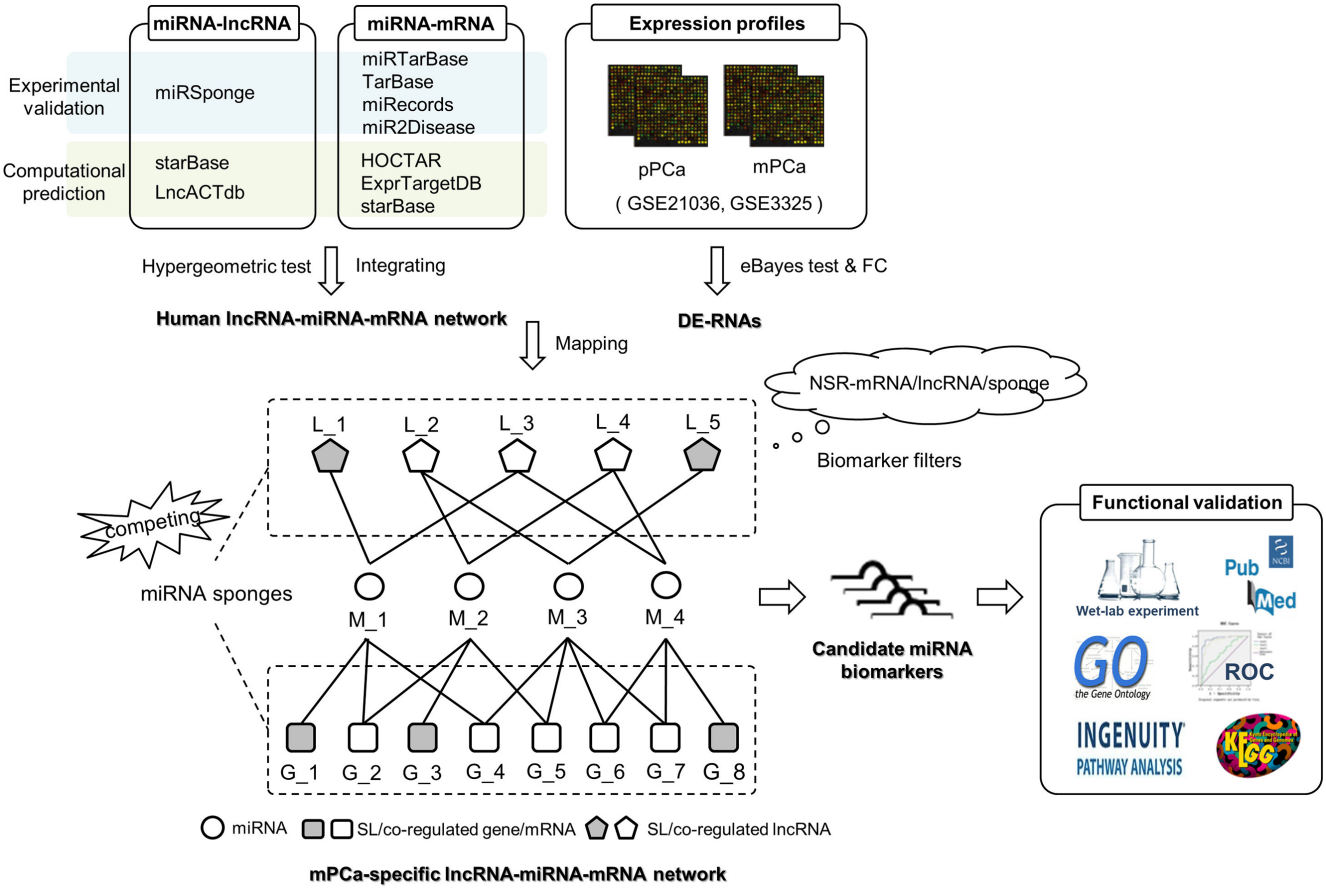
The schematic pipeline of this study. pPCa: primary prostate cancer; mPCa: metastatic prostate cancer; FC: fold change; DE: differentially expressed; NSR: number of single-line regulations; SL: single-line; GO: gene ontology; ROC: receiver operating characteristic curve; KEGG: Kyoto Encyclopedia of Genes and Genomes.

The existing findings indicate the significance of ceRNAs in cancer biology, and the modeling of deregulated ceRNA networks promotes a novel understanding of miRNA-mediated pathogenesis in cancers. However, few of the methods focus on the hidden structures in the network, e.g. vulnerable regulatory sites, etc., and there are no general rules for biomarker discovery. In our previous work, an evidence-based bioinformatics model was proposed and applied to identify miRNA biomarkers for PCa metastasis by measuring the number of single-line regulations (NSR) between miRNAs and mRNAs in the miRNA–mRNA network.^12^, ^13^ In the model, miRNAs with strong single-line regulatory power, i.e. number of genes independently regulated by a single miRNA, were statistically important and could serve as candidate biomarkers. In this study, we improved the computational framework by comprehensively characterizing the regulatory role of miRNAs competed by lncRNAs and mRNAs, and extracted novel topological feature parameters as prior evidence for miRNA biomarker discovery in the lncRNA–miRNA–mRNA ceRNA network. Then the model was applied to identify novel miRNA biomarkers for predicting PCa metastasis. The schematic pipeline is shown in Fig. 1.

## Materials and methods

### Data collection and integration

The literature-reported cancer miRNA biomarkers were manually collected from published papers in PubMed using search criteria “(miRNA*[tiab] OR microRNA*[tiab]) AND (biomarker*[tiab] OR marker*[tiab] OR indicator*[tiab] OR predict*[tiab])”. The miRNA symbols were normalized based on the records in miRBase.^14^

The miRNA-targets regulatory data were integrated from public databases and software tools. Among them, miRNA–mRNA were derived from our previous study,^12^ where experimentally validated and computationally predicted miRNA–mRNA interactions were merged and analyzed. The miRNA–lncRNA pairs were downloaded from miRSponge,^15^ LncACTdb,^5^ and starBase.^16^ Here human miRNA–lncRNA regulations and genome data were selected and the number of supporting experiments in starBase was set as ≥1. In addition, functional genes including transcription factor (TF) genes,^17^ essential genes,^18^ house-keeping genes,^19^, ^20^ and tumor-associated genes (i.e. oncogenes, tumor suppressor genes, and marker genes)^21^, ^22^ were extracted from public databases and the literature, respectively, to evaluate the biological functions of different miRNAs in gene regulation.

To better compare the predictive performance of bioinformatics models constructed in this study and our previous work,^13^ the same publicly available datasets from the GEO (Gene Expression Omnibus) database were selected for gene expression analysis.^23^ Here GSE21036 contained a total of 142 prostate tissue samples, and 99 primary prostate cancer (pPCa) and 14 metastatic prostate cancer (mPCa) samples were chosen for further study.^24^ GSE3325 includes two types of sample data, i.e. individual samples and pooled samples. In this study 5 and 4 individual samples for pPCa and mPCa respectively were analyzed.^25^ The differentially expressed (DE) miRNAs and mRNAs were screened using the empirical Bayes method in the R package of Linear Models for Microarray Data Analysis,^26^ and the raw *P*-values were adjusted based on the Benjamini–Hochberg false discovery rate method. The threshold for DE-RNA extraction was set as adjusted *P*-value (adj.*P*-value) < 0.05 and |log_2_FC| > 1.

### Construction of condition-specific lncRNA–miRNA–mRNA network

Based on the “ceRNA hypothesis”, a three-step approach was applied to construct the mPCa-specific lncRNA–miRNA–mRNA network. First, the miRNA–mRNA and miRNA–lncRNA pairs were identified respectively, based on the collected miRNA regulatory data on mRNAs and lncRNAs. Then, a human global lncRNA–miRNA–mRNA network was constructed as the reference if miRNA–mRNA and miRNA–lncRNA pairs significantly shared common miRNAs. The statistical significance was calculated using a hypergeometric test, followed by Benjamini–Hochberg correction for raw *P*-value adjustment. All of the triplets with adj.*P*-value < 0.01 were selected and integrated. Finally, DE-miRNAs and DE-mRNAs between pPCa and mPCa samples were mapped onto the reference network and the mPCa-specific lncRNA–miRNA–mRNA network together with functional lncRNAs were extracted and inferred for computational modelling.

### Novel evidence-based bioinformatics model for miRNA biomarker discovery

The single-line regulatory ability of miRNAs was characterized and measured in our previous work based on miRNA–mRNA network analyses since single-line regulation is the vulnerable site in the network compared with the traditional “multiple-to-multiple” pattern between miRNAs and mRNAs.^12^ According to statistical evidence, miRNAs with stronger single-line regulatory power (quantified as higher NSR values) are more likely to be biomarkers for disease prediction. Based on this principle, as shown in Fig. 1, single-line regulation is also evaluated in the constructed lncRNA–miRNA–mRNA network. Hence in this study the network characterization was improved by reasonably weighting the single-line regulatory and competing mechanisms among miRNAs, lncRNAs, and mRNAs. Three parameters, i.e. NSR-mRNA, NSR-lncRNA, and NSR-sponge, were defined to describe the number of single-line regulations of miRNAs on mRNAs, lncRNAs, and sponges, respectively. Herein, NSR-sponge = NSR-mRNA + NSR-lncRNA. Finally, miRNAs with significantly high NSR-mRNA, NSR-lncRNA, and NSR-sponge values (*P*-value < 0.05, Wilcoxon signed-rank test) were identified as candidate biomarkers.

### Computational validation and functional survey

Receiver operating characteristic (ROC) analysis was conducted based on the expression data of identified miRNAs to evaluate their abilities in differentiating mPCa samples from pPCa using the R package “epicalc”. Functional analyses, including GO (Gene Ontology) annotation (i.e. biological process, BP; cellular component, CC; molecular function, MF), KEGG (Kyoto Encyclopedia of Genes and Genomes) pathway enrichment and integrated IPA (Ingenuity Pathway Analysis) analysis, were performed on the targets of identified miRNAs using the online tools DAVID (Database for Annotation, Visualization and Integrated Discovery) and IPA.^27–29^ Here the targets of identified miRNAs were derived from human miRNA–mRNA pairs and the plug-in EnrichmentMap in the Cytoscape program was applied for functional clustering of significantly enriched BP terms.

### Cell lines and culture conditions

The human PCa cell lines 22RV1, LNCaP, PC3, and DU145 as well as human non-tumor prostatic cell line WPMY-1 were obtained from the Cell Bank of Chinese Academy of Sciences (Shanghai, China). Among them, 22RV1 and LNCaP cells were cultured in RPMI-1640 medium (Thermo Fisher Scientific, USA) supplemented with 10% fetal bovine serum (FBS) (Thermo Fisher Scientific, USA), 1% glutamax (Thermo Fisher Scientific, USA), and 1 mM sodium pyruvate (Thermo Fisher Scientific, USA); PC-3 cells were cultured in Ham's F-12K (Kaighn's) medium (Thermo Fisher Scientific, USA) supplemented with 10% FBS (Thermo Fisher Scientific, USA); DU145 cells were cultured in MEM medium (Thermo Fisher Scientific, USA) supplemented with 10% FBS (Thermo Fisher Scientific, USA), 1% glutamax (Thermo Fisher Scientific, USA), 1% non-essential amino acids (Thermo Fisher Scientific, USA) and 1 mM sodium pyruvate (Thermo Fisher Scientific, USA); WPMY-1 cells were cultured in DMEM medium (Thermo Fisher Scientific, USA) containing 10% FBS (Thermo Fisher Scientific, USA). To inhibit the growth of bacteria, the medium was also supplemented with 100 U/mL penicillin and 100 μg/mL streptomycin (Thermo Fisher Scientific, USA), respectively. All cell lines were incubated at 37°C in a 5% CO_2_ atmosphere.

### qRT-PCR experiment and statistical analysis

In the experiment, total RNA from the cultured cells was isolated using an miRNeasy MiniKit (Qiagen, USA) and then reverse transcribed to complementary DNA (cDNA) utilizing an miScript II RT Kit (Qiagen, USA). To determine the expression level of miRNAs of interest, qRT-PCR assay was performed on the Roche LightCycler 480 Real-Time PCR system (Roche, USA) using a miScript SYBR Green PCR Kit (Qiagen, USA) according to the manufacturers’ protocols. The primer sequences used in qRT-PCR are summarized in Table S1, see online supplementary material. Each qRT-PCR experiment was conducted in triplicate. The relative miRNA expression level was quantified by the 2^−ΔΔCt^ method using snRNA U6 as the reference.

## Results

### Comparison of miRNA regulation on mRNAs and lncRNAs

In this study miRNA–mRNA and miRNA–lncRNA networks were constructed respectively. The miRNA–mRNA network included a total of 48 868 interactions among 618 miRNAs and 9 526 mRNAs, whereas there were 11 893 regulatory pairs among 307 miRNAs and 1 296 lncRNAs in the miRNA–lncRNA network. The regulatory roles of miRNAs on mRNAs and lncRNAs were analyzed and compared based on the constructed binary networks. As shown in Fig. S1, see online supplementary material, in the miRNA–mRNA network miRNA with more mRNA targets (>200) were few in number, however, all of these miRNAs were able to regulate lncRNAs. There was a significantly positive correlation between the number of mRNAs and lncRNAs targeted by miRNAs (*r* = 0.62, *P*-value < 2.2e−16, Spearman correlation test). Furthermore, only a few miRNAs in the miRNA–mRNA network had stronger single-line regulatory power (≥10), and most of these miRNAs tended to have lncRNA targets. The number of lncRNA targets was also positively correlated with the single-line regulatory power of miRNAs on mRNAs (*r* = 0.43, *P*-value < 2.2e−16, Spearman correlation test).

**Figure 2.**
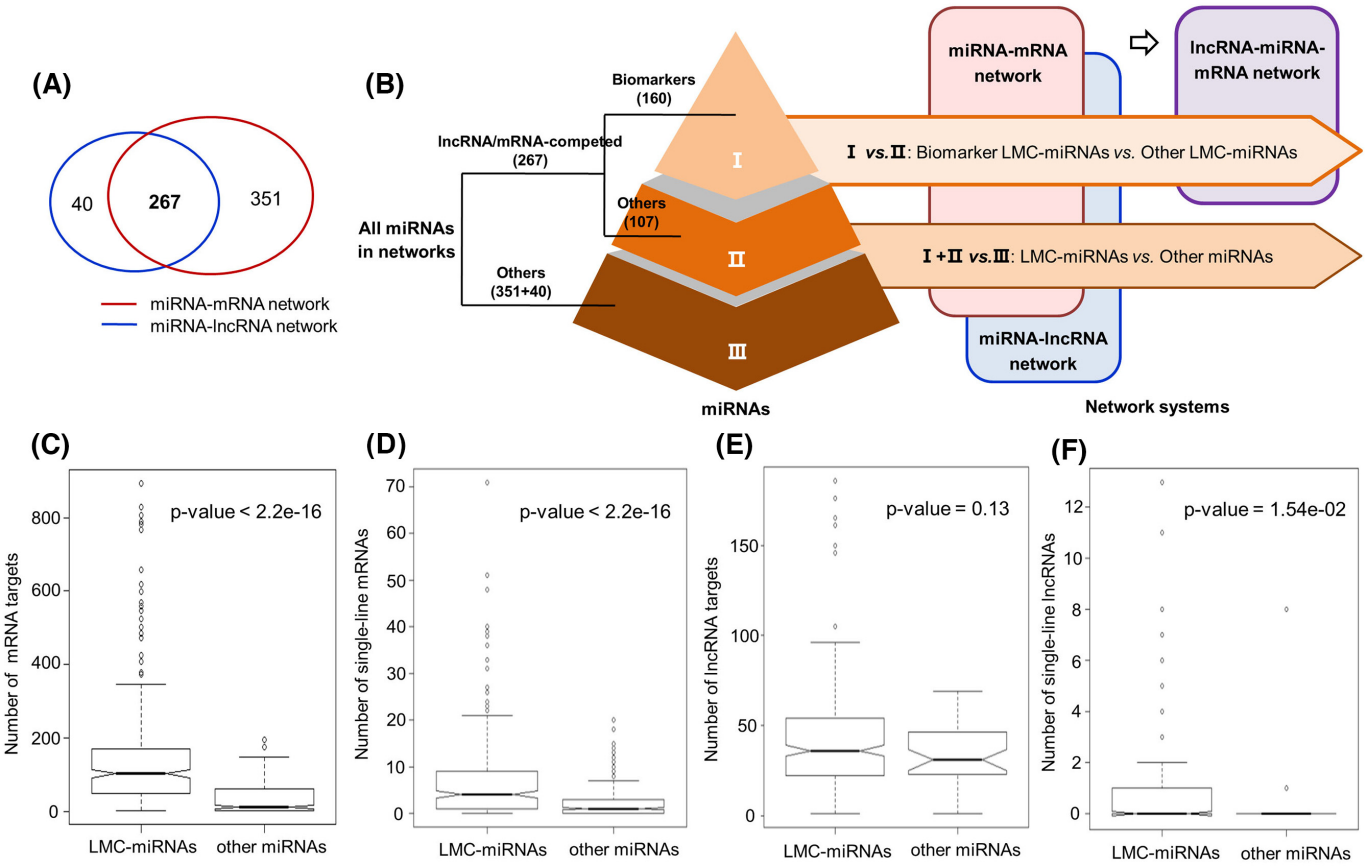
Comparison of miRNA regulation on mRNAs and lncRNAs. (**A**) A total of 267 miRNAs shared by the miRNA–mRNA and miRNA–lncRNA networks were defined as LMC-miRNAs. (**B**) Schematic workflow for miRNA classification. First, all miRNAs were partitioned into LMC-miRNAs and other miRNAs. Then, reported biomarker miRNAs were extracted from LMC-miRNAs for further analysis. (**C**) In the miRNA–mRNA network, LMC-miRNAs had stronger regulatory power on mRNAs (*P*-value < 2.2e−16). (**D**) In the miRNA–mRNA network, LMC-miRNAs had stronger single-line regulatory power on mRNAs (*P*-value < 2.2e−16). (**E**) In the miRNA–lncRNA network, there was no significant difference in the number of lncRNA targets between LMC-miRNAs and other miRNAs (*P*-value = 0.13). (**F**) In the miRNA–lncRNA network, LMC-miRNAs had stronger single-line regulatory power on lncRNAs (*P*-value = 1.54e−02). The statistical significance was calculated using Kolmogorov-Smirnov test. LMC-miRNAs: lncRNA/mRNA-competed miRNAs.

As shown in Fig. 2, a total of 267 miRNAs shared by the two network systems were classified and extracted, which indicated that they could be potentially competed by mRNAs and lncRNAs. These miRNAs were defined as lncRNA/mRNA-competed miRNAs (LMC-miRNAs). Compared with other miRNAs, LMC-miRNAs could regulate more mRNAs, and they also had significantly stronger single-line regulatory power (*P*-value < 2.2e−16, Kolmogorov–Smirnov test) in the miRNA–mRNA network. In the miRNA–lncRNA network, there was no significant difference in the number of lncRNA targets between LMC-miRNAs and other miRNAs. However, the single-line regulatory power of LMC-miRNAs on lncRNAs was significantly higher than other miRNAs. In terms of biological functions, a total of 1 834 TF genes, 2 570 essential genes, 575 house-keeping genes and 1 764 tumor-associated genes were respectively collected from public databases and the literature. As shown in Fig. S2, see online supplementary material, the percentage of these genes in LMC-miRNA targets was significantly higher than that of other miRNAs, indicating the functional importance of LMC-miRNAs in cancer-related biological pathogenesis.

In conclusion, there was a positive correlation between miRNA regulation on mRNAs and lncRNAs. Compared with other miRNAs, LMC-miRNAs had stronger (single-line) regulatory power, and they could regulate more functional genes. Thus, it is of significance to integrate the multivariate regulatory data and construct a lncRNA–miRNA–mRNA network for cancer miRNA biomarker discovery.

### Feature characterization of reported biomarkers in miRNA-mediated network systems

**Figure 3.**
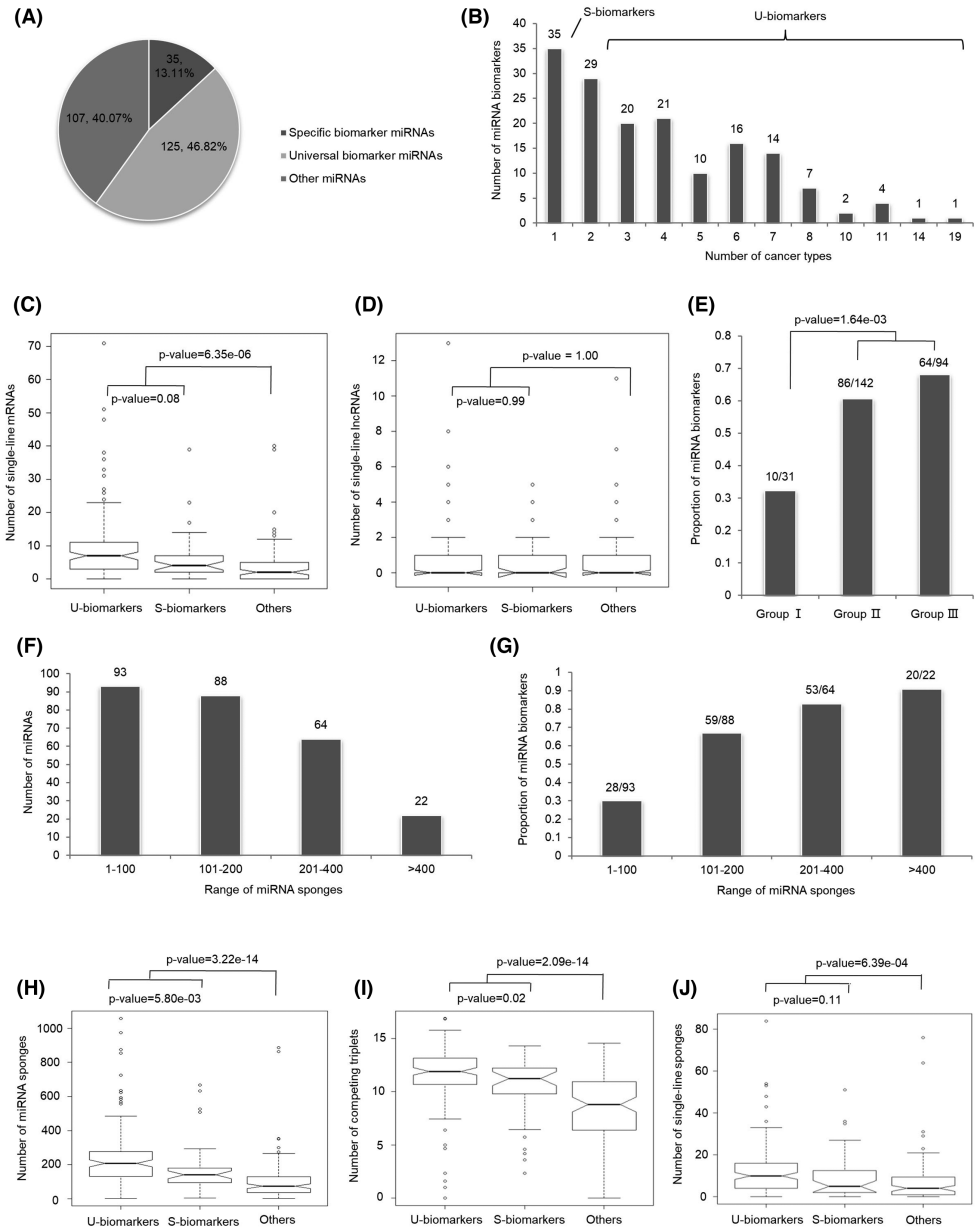
Topological characterization of reported miRNA biomarkers in the networks. (**A, B**) Distribution of literature-reported biomarkers in the set of LMC-miRNAs. (**C, D**) Comparison of the single-line regulatory power of different miRNAs on mRNAs and lncRNAs. (**E**) Proportion of biomarker miRNAs in three groups. Here “10/31” represents that there were 31 miRNAs in this group, and 10 of them were reported biomarkers. The statistical significance was calculated using Pearson's chi-squared test. Group I: miRNAs with no single-line regulatory power. Group II: miRNAs with single-line regulatory power solely on mRNAs or lncRNAs. Group III: miRNAs with single-line regulatory power on both mRNAs and lncRNAs. (**F**) miRNAs with more sponges (mRNAs and lncRNAs) in the lncRNA–miRNA–mRNA network were fewer in number. (**G**) Biomarker miRNAs tended to have more sponges. Here “28/93” represents that there were 93 miRNAs with 1–100 sponges in the network, and 28 of them were biomarkers. (**H**) Comparison of the number of miRNA sponges between biomarker miRNAs and other miRNAs. (**I**) Comparison of the number of completing triplets between biomarker miRNAs and other miRNAs. The number was log-2 base transformed. Here the number of completing triplets associated with a certain miRNA was positively correlated with its sponge number (*r* = 0.94, *P*-value < 2.2e−16, Spearman correlation test). (**J**) Comparison of the number of single-line sponges between biomarker miRNAs and other miRNAs. The statistical significance was calculated using the Kolmogorov–Smirnov test. S-biomarkers: specific biomarkers; U-biomarkers: universal biomarkers.

As described in Table S2, see online supplementary material, a total of 160 literature-reported miRNA biomarkers were manually collected from 789 published papers in PubMed. According to “miRNA-cancer” associations, as shown in Figs. 3A and 3B, all of the biomarker miRNAs were classified into two groups, i.e. miRNAs related to more than one cancer type (universal biomarkers, U-biomarkers) and miRNAs specific to only one cancer type (specific biomarkers, S-biomarkers).

As analyzed above, miRNAs competed by lncRNAs and mRNAs (LMC-miRNAs) were both structurally and functionally important in the network. Based on this finding, the regulatory power of biomarker miRNAs and other miRNAs were compared in the miRNA–mRNA, miRNA–lncRNA, and integrated lncRNA–miRNA–mRNA networks, respectively. As shown in Figs. 3C and 3D, compared with other miRNAs, biomarker miRNAs tended to have stronger single-line regulatory power on mRNAs. However, there was no significant difference in the number of single-line regulated lncRNAs between biomarker miRNAs and others. Based on the characterization of single-line regulation, as shown in Fig. 3E, miRNAs were further divided into three groups, i.e. miRNAs with no single-line regulatory power (Group I), miRNAs with single-line regulatory power solely on mRNAs or lncRNAs (Group II), and miRNAs with single-line regulatory power on both mRNAs and lncRNAs (Group III). The result indicated that miRNAs with stronger single-line regulatory power were likely to be biomarkers (Group III > Group II > Group I).

As shown in Figs. 3F and 3G, there were several miRNAs competed by a large number of lncRNAs and mRNAs (termed miRNA sponges) in the lncRNA–miRNA–mRNA network. Although the number of these miRNAs was relatively small, most of them were literature-reported biomarkers. As illustrated in Figs. 3H and 3I, biomarker miRNAs tended to have a high degree of centrality in the network, and they could mediate more competing triplets compared with other miRNAs. In particular, as shown in Fig. 3J, biomarker miRNAs also had significantly strong single-line regulatory power in the lncRNA–miRNA–mRNA network and they could be independently competed by lncRNAs and mRNAs, which provided the theoretical evidence for ceRNA-based biomarker discovery.

### Bioinformatics model for miRNA biomarker discovery: a case study of PCa metastasis

The human global lncRNA–miRNA–mRNA network contained 1 381 492 triplets among 267 miRNAs, 1 280 lncRNAs and 8 392 mRNAs. By mapping miRNAs and mRNAs differentially expressed between pPCa and mPCa groups, an mPCa-specific lncRNA–miRNA–mRNA network with 12 167 deregulated triplets among 57 DE-miRNAs, 247 DE-mRNAs, and 808 functional lncRNAs was extracted. As listed in Table S3, see online supplementary material, a total of 12 miRNAs with significantly high NSR-mRNA, NSR-lncRNA, and NSR-sponge value (*P*-value < 0.05, Wilcoxon signed-rank test) were identified as candidate biomarkers for PCa metastasis based on the defined filters.

As illustrated in Table 1, the identified miRNAs were all down-regulated in mPCa groups compared with pPCa. The ROC analysis indicated the potential of identified miRNAs for differentiating pPCa and mPCa samples with an average area under the curve (AUC) value of 0.8707 (ranging from 0.7489 to 0.9928). Among them, three miRNAs, i.e. *miR-204-5p, miR-145-5p*, and *miR-101-3p*, were consistent with the result of our previous model solely using miRNA–mRNA data for network characterization.^13^

**Table 1.** Candidate miRNA biomarkers identified for PCa metastasis.

miRNA ID	adj.*P*-value (pPCa vs. mPCa)	log_2_ (FC)	NSR-mRNA (*P*-value)	NSR-lncRNA (*P*-value)	NSR-sponge (*P*-value)	AUC
*miR-23b-3p*	2.76e-16	−1.9452	9 (4.02e-16)	21 (8.52e-13)	30 (1.43e-14)	0.9567
*miR-204-5p*	3.43e-08	−2.0896	13 (1.39e-17)	12 (4.25e-08)	25 (2.14e-12)	0.8506
*miR-26b-5p*	2.75e-07	−1.6402	10 (1.25e-16)	15 (1.91e-11)	25 (2.14e-12)	0.7489
*miR-27b-3p*	1.22e-14	−1.7558	12 (2.78e-17)	12 (4.25e-08)	24 (2.19e-12)	0.9574
*miR-145-5p*	8.00e-25	−3.2157	4 (5.89e-06)	16 (7.45e-12)	20 (7.79e-11)	0.9928
*miR-29b-3p*	4.49e-06	−1.0465	4 (5.89e-06)	14 (1.60e-10)	18 (1.44e-09)	0.7468
*miR-143-3p*	9.38e-29	−4.0419	3 (4.25e-04)	14 (1.60e-10)	17 (1.21e-08)	0.9942
*miR-130a-3p*	2.43e-17	−1.9649	3 (4.25e-04)	12 (4.25e-08)	15 (1.41e-06)	0.9365
*miR-363-3p*	4.45e-08	−2.0687	4 (5.89e-06)	11 (9.37e-07)	15 (1.41e-06)	0.7792
*miR-218-5p*	7.87e-11	−1.6984	4 (5.89e-06)	9 (1.14e-03)	13 (1.92e-04)	0.8709
*miR-30c-5p*	2.16e-07	−1.0445	4 (5.89e-06)	9 (1.14e-03)	13 (1.92e-04)	0.8225
*miR-101-3p*	1.26e-08	−1.0684	3 (4.25e-04)	8 (2.07e-02)	11 (1.16e-02)	0.7915

adj.*P*-value: adjusted *P*-value; pPCa: primary prostate cancer; mPCa: metastatic prostate cancer; FC: fold change; NSR: number of single-line regulation; AUC: area under the curve.

Based on literature validation, all 12 miRNAs were associated with PCa carcinogenesis, and 9 of them (75%, 9/12, i.e. *miR-23b-3p, miR-204-5p, miR-27b-3p, miR-145-5p, miR-29b-3p, miR-143-3p, miR-218-5p, miR-30c-5p*, and *miR-101-3p*) had been already reported as biomarkers for PCa metastasis, demonstrating the overall prediction precision and the outperformance of the proposed model compared with our previous approach (40%).^13^ For example, Rice *et al*. found that the cluster of *miR-23b/-27b* could suppress PCa metastasis by decreasing the level of *HIP1R* in PCa pre-clinical models.^30^ Compared with PCa samples without bone metastasis, Wa *et al*. identified that *miR-204-5p* was down-regulated in PCa groups with bone metastasis, and it could inactivate *NF-κB* signaling to inhibit the process of PCa metastasis.^31^  *miR-145-5p* and *miR-143-3p* are two well-studied miRNAs in PCa progression. The former is an important tumor suppressor miRNA that plays functional roles in controlling oncogenes such as *MYC* and *RAS* in PCa development. Iscaife *et al*. reported that *miR-145* was a promising biomarker for the treatment of metastatic castration-resistant PCa.^32^ Moreover, the panel of *miR-143* and *miR-145* was a significant signature for discriminating different stages of PCa and indicating bone metastasis.^33^ Ru *et al*. suggested that *miR-29b* was an antimetastatic miRNA for PCa cells, and it could suppress PCa metastasis by targeting epithelial–mesenchymal transition signaling.^34^ Leite *et al*. reported that the alteration in expression of *miR-218* was associated with the progression of PCa from high-grade localized PCa to metastasis status.^35^ Ren *et al*. performed comprehensive miRNA microarray analysis and *miR-30c* was found to be significantly down-regulated in the metastatic stage of PCa.^36^ The reduced level of *miR-101* could suppress the expression of the master regulators in PCa metastasis, i.e. *FOXM1* and *CENPF*, which confirmed its contribution in the cascade responses in PCa development. Although the remaining three miRNAs (i.e. *miR-26b-5p, miR-130a-3p*, and *miR-363-3p*) have not been reported as biomarkers for mPCa, they were shown to be functional in PCa genesis.^37–39^ Thus, the predictive power of these miRNAs in PCa metastasis needs to be further validated and explored.

### Functional enrichment analyses and cell line experimental validation

The GO and pathway enrichment analyses were performed based on the targets of identified miRNA biomarkers. As shown in Table S4 and Fig. S3 (see online supplementary material), the miRNAs were involved in PCa-related GO terms, and significantly enriched BP terms were clustered as cell migration, cell cycle, molecular transport, and cell development, which highlighted the underlying mechanisms of identified miRNAs in PCa pathogenesis.^40^

**Figure 4.**
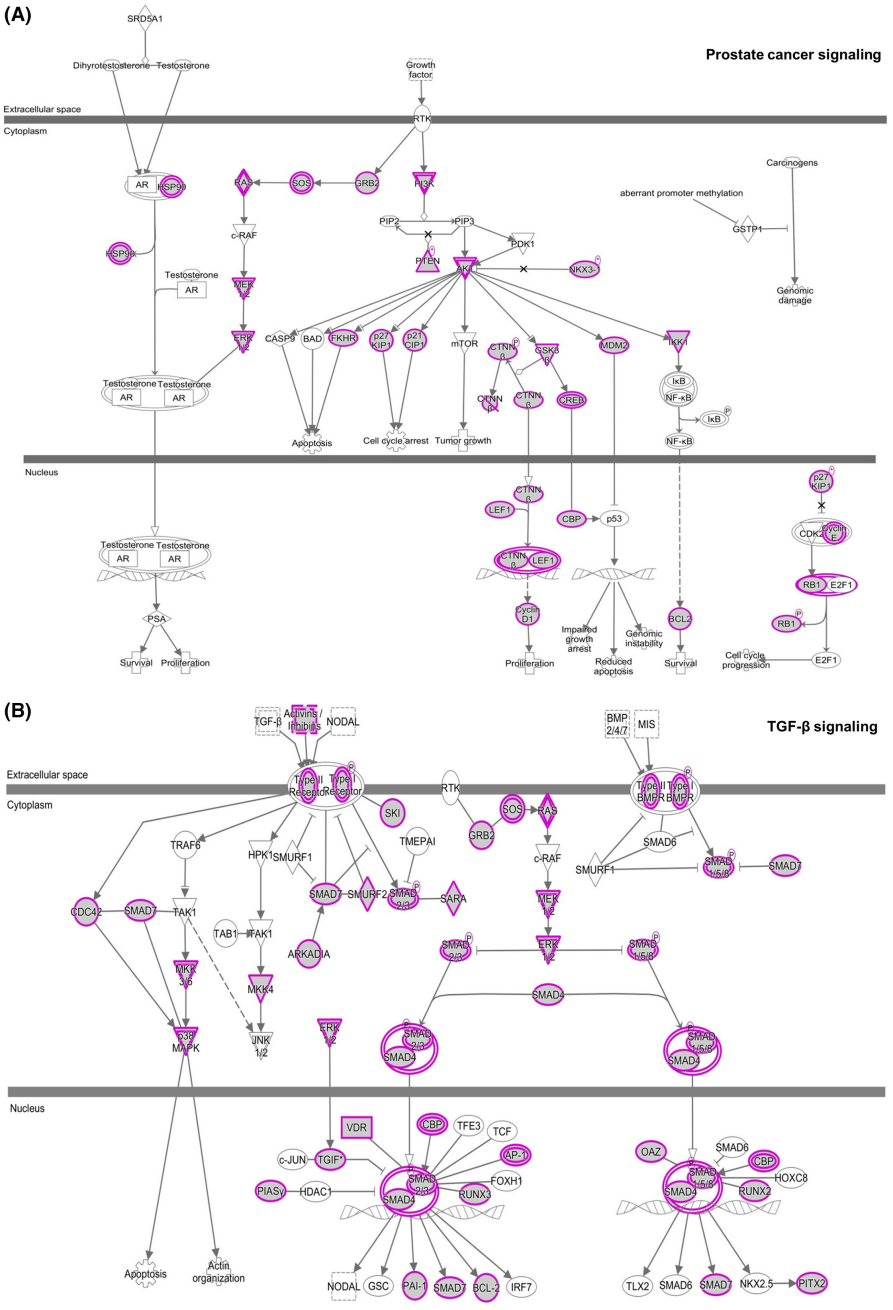
Significantly enriched PCa-related pathways. (**A**) Prostate cancer signaling. (**B**) *TGF-β* signaling. The figures were extracted from the IPA program. Objects with purple circles or triangles were acting loci by mapped genes.

For pathway enrichment, as shown in Table S5 (see online supplementary material), the targets of identified miRNAs were closely enriched in pathways associated with PCa evolution,^41–43^ e.g. prostate cancer, cell cycle, *p53* signaling, *ERK/MAPK* signaling, *PI3K/AKT* signaling, etc. As illustrated in Fig. 4, taking prostate cancer signaling and *TGF-β* signaling as examples, most of the miRNA targetswere located in the hub sites of these pathways, e.g. *PI3K, AKT, ERK1/2, SMAD4, SMAD* family, etc. Among them, in prostate cancer signaling, the genes regulated by identified miRNAs were functional in mediating cell proliferation, apoptosis, survival, and cell cycle arrest during PCa progression. In *TGF-β* signaling, the Type Ⅰ/Ⅱ receptors and Type Ⅰ/Ⅱ *BMPR* with essential roles in transferring *TGF-β* signal from the extracellular space to the cytoplasm were potentially targeted by identified miRNAs, which inferred the latent pathogenesis of these miRNAs in *TGF-β*-related processes of PCa metastasis.^44^, ^45^

**Figure 5.**
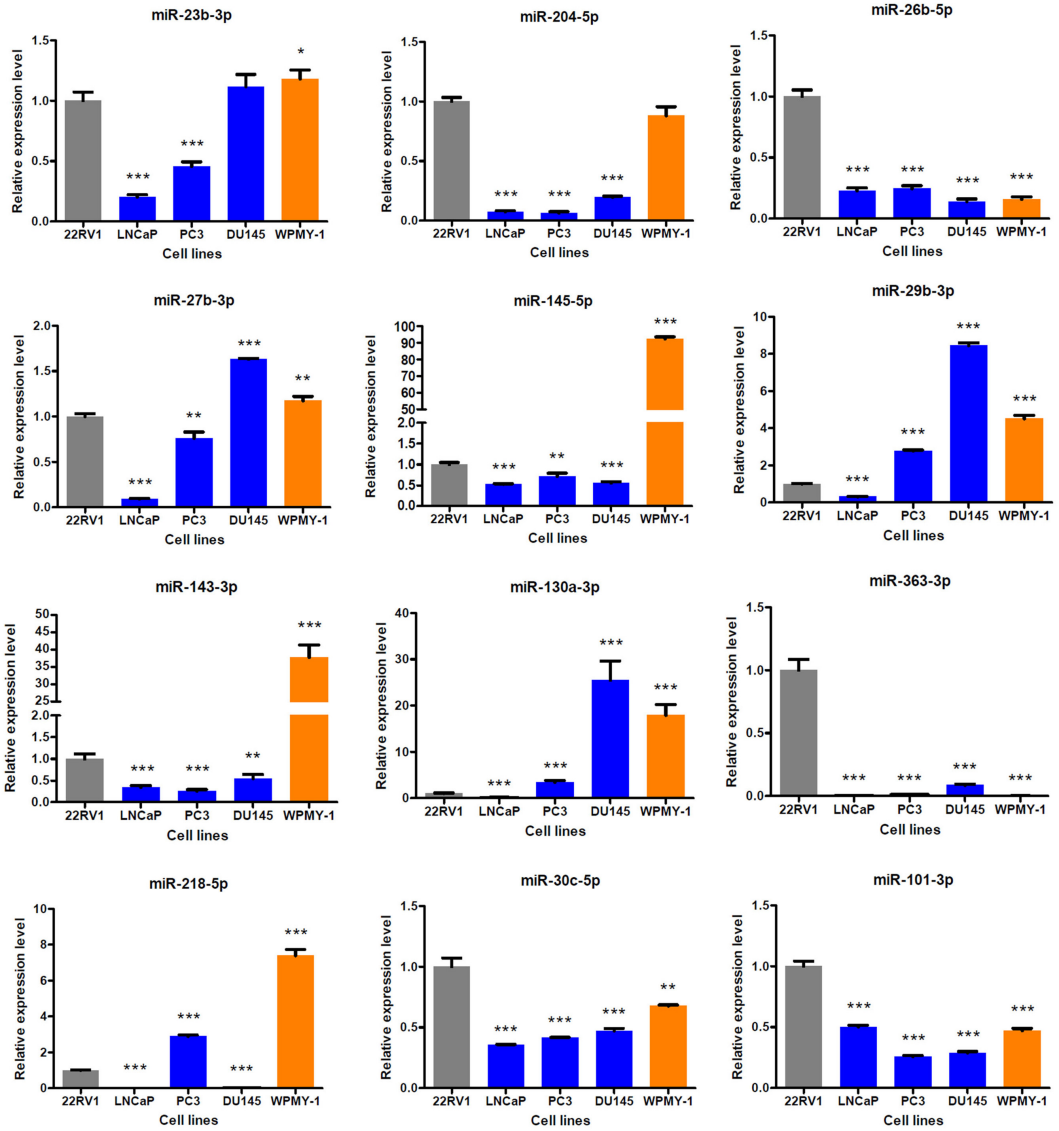
qRT-PCR validation of identified miRNA biomarkers using cell line samples (2^–ΔΔCt^ method). Here 22RV1 was a pPCa-related cell line, whereas LNCaP, PC3, and DU145 were cell lines for mPCa. In addition, WPMY-1 was a normal prostate cell line. *: *P*-value < 0.05; **: *P*-value < 0.01; ***: *P*-value < 0.001 (compared with 22RV1).

The qRT-PCR experiment was conducted using PCa cell line samples to evaluate the expression alternation of identified miRNAs between pPCa and mPCa cell line groups. As shown in Fig. 5, most of the miRNAs were significantly deregulated in mPCa cell lines including LNCaP, PC3, and DU145 compared with pPCa cell line 22RV1. Among them, the expression of seven miRNAs, i.e. *miR-204-5p, miR-26b-5p, miR-145-5p, miR-143-3p, miR-363-3p, miR-30c-5p*, and *miR-101-3p*, was significantly decreased (*P*-value < 0.01 or *P*-value < 0.001) in all the three mPCa cell lines, which indicated the suppressing role of these miRNAs in PCa metastasis. In addition, *miR-27b-3p, miR-29b-3p, miR-130a-3p*, and *miR-218-5p* were found to be differentially expressed in mPCa groups (*P*-value < 0.01 or *P*-value < 0.001), however, their expression pattens in different cell lines tended to be heterogeneous. The expression level of *miR-23b-3p* in LNCaP and PC3 cells was decreased, but the significance of this was not observed in the DU145 cell line. Most of the identified miRNAs were also shown to be significantly deregulated between 22RV1 and the normal prostate cell line WPMY-1, suggesting the functional importance of these miRNAs in PCa occurrence.

Overall, the experimental result was highly consistent with that in bioinformatics analysis, which demonstrated the biomarker potential of identified miRNAs in PCa carcinogenesis. In particular, three miRNAs, i.e. *miR-26b-5p, miR-130a-3p*, and *miR-363-3p*, were confirmed to be novel candidates for mPCa, which highlighted the predictive power of the proposed model in translational PCa study.

## Discussion

The metastasis of PCa is life-threatening and leads to poor prognosis and short overall survival of patients. The identification and prioritization of biomarkers with high sensitivity for precise detection of PCa metastatic signatures are therefore of clinical significance. In addition to experiment-guided approaches, computer-aided biomarker discovery based on multi-omics data integration and network modeling is becoming the current frontier in bioinformatics and medical systems biology, which contributes to systematic understanding of cancer carcinogenesis in the era of big biomedical data and translational informatics.

In this study a novel bioinformatics model was constructed based on the “ceRNA hypothesis” and topological characterization of the miRNA-mediated lncRNA–miRNA–mRNA network. Compared with traditional methods solely focusing on hub property in the network,^46^, ^47^ in this model single-line regulation and competition among miRNAs, lncRNAs, and mRNAs were measured for biomarker discovery, since the single-line mode is vulnerable in the network and deregulation in such sites would cause disorder at the systems level. Based on our previous findings, miRNAs with stronger single-line regulatory power on mRNAs in the miRNA–mRNA network were likely to be biomarkers.^12^ Hence this special structural feature was analyzed in the lncRNA–miRNA–mRNA network to highlight potential clues for ceRNA-based screening of miRNA biomarkers for cancer management. According to statistical evidence, miRNAs competed by lncRNAs and mRNAs had stronger regulatory power than other miRNAs, and those with a large number of single-line targets, i.e. significant NSR-mRNA/lncRNA/sponge value, tended to have a high possibility of serving as cancer biomarkers.

The application of the proposed bioinformatics model identified a total of 12 miRNAs as candidate biomarkers for PCa metastasis, i.e. *miR-23b-3p, miR-204-5p, miR-26b-5p, miR-27b-3p, miR-145-5p, miR-29b-3p, miR-143-3p, miR-130a-3p, miR-363-3p, miR-218-5p, miR-30c-5p*, and *miR-101-3p*. Among them, 9 miRNAs (75%, 9/12) were reported as mPCa biomarkers based on PubMed literature mining. Overall, the predictive precision outperformed our previous approach and ROC analysis confirmed the power of identified miRNAs for classifying pPCa and mPCa samples. Moreover, a low-throughput experiment using a prostate cell line and qRT-PCR method indicated the potential of *miR-26b-5p, miR-130a-3p*, and *miR-363-3p* as novel biomarkers for prediction of PCa metastasis. Functional analyses demonstrated underlying carcinogenesis of identified miRNAs as well as competing interactions in mediating PCa-associated pathways such as prostate cancer signaling, *p53* signaling, *ERK/MAPK* signaling, and *TGF-β* signaling.

It should be noted that shortcomings and limitations in this study still need to be carefully considered and addressed. First, the structural features of different types of RNA nodes were characterized in the ceRNA-based network, and with the accumulation of biological data the analytical strategy should be improved by reasonably adding new parameters associated with the functional importance of RNAs during PCa progression and metastasis. Second, in the current model only lncRNAs and mRNAs were considered as ceRNA components for network characterization, however, according to the “ceRNA hypothesis” circular RNAs (circRNAs) and pseudogenes also contributed to the competition on miRNAs for down-stream gene regulation, hence the network system needs to be expanded for multivariate detection of biomarkers at multi-RNA levels, e.g. lncRNAs, circRNAs, mRNAs as well as their combined or module signatures. Last but most important, functional validation and a pathogenic survey using human samples and clinical data will be conducted in our next-step work for further translational research of the findings.

## Supplementary Material

pbac001_Supplemental_Tables_and_Figures
