# Supplementary material for: Multivariate competing endogenous RNA network characterization for cancer microRNA biomarker discovery: a novel bioinformatics model with application to prostate cancer metastasis
Source: Precis Clin Med. 2022 Jan 10;5(1):pbac001. doi: 10.1093/pcmedi/pbac001 (PMC9267254; doi:10.1093/pcmedi/pbac001)
Supplement: pbac001_Supplemental_Tables_and_Figures [file pbac001_supplemental_tables_and_figures.zip › Figure-S1.doc]

**Figure S1. Relationship between miRNA regulation on mRNAs and lncRNAs.**

**A.** In miRNA-mRNA network, miRNAs with more mRNA targets were fewer in number. **B.** miRNAs with more mRNA targets were powerful to regulate lncRNAs. Here ‘69/318’ represents that there were 318 miRNAs with 1-50 mRNA targets in miRNA-mRNA network, and 69 of them had lncRNA targets according to the association in miRNA-lncRNA network. **C.** The number of lncRNA targets was positively correlated with the number of mRNA targets (r=0.62, p-value < 2.2e-16, Spearman correlation test). **D.** miRNAs with stronger single-line regulatory power (higher NSR values) were fewer in miRNA-mRNA network. **E.** miRNAs with higher NSRs held the potential to target lncRNAs. Here ‘41/208’ represents that there were 208 miRNAs with NSR=0 in miRNA-mRNA network, and 41 of them had lncRNA targets. **F.** The number of lncRNA targets was positively correlated with their single-line regulatory power (r=0.43, p-value < 2.2e-16, Spearman correlation test).

**Abbreviation:** NSR: number of single-line regulation.
