# Supplementary material for: Multivariate competing endogenous RNA network characterization for cancer microRNA biomarker discovery: a novel bioinformatics model with application to prostate cancer metastasis
Source: Precis Clin Med. 2022 Jan 10;5(1):pbac001. doi: 10.1093/pcmedi/pbac001 (PMC9267254; doi:10.1093/pcmedi/pbac001)
Supplement: pbac001_Supplemental_Tables_and_Figures [file pbac001_supplemental_tables_and_figures.zip › Figure-S2.doc]

**Figure S2. Comparison of functional characteristics between LMC-miRNAs and other miRNAs.**

**A, B, C,** and **D.** Proportion of transcription factor genes, essential genes, house-keeping genes, and tumor-associated genes targeted by miRNAs in miRNA-mRNA network. The statistical significance was calculated using Kolmogorov-Smirnov test.

**Abbreviation:** LMC-miRNAs: lncRNA/mRNA-competed miRNAs.
