# Supplementary material for: Multivariate competing endogenous RNA network characterization for cancer microRNA biomarker discovery: a novel bioinformatics model with application to prostate cancer metastasis
Source: Precis Clin Med. 2022 Jan 10;5(1):pbac001. doi: 10.1093/pcmedi/pbac001 (PMC9267254; doi:10.1093/pcmedi/pbac001)
Supplement: pbac001_Supplemental_Tables_and_Figures [file pbac001_supplemental_tables_and_figures.zip › Figure-S3.doc]

**Figure S3. Clustering for significantly enriched BP terms based on GO annotation.**

Here each node represents a BP term and it was clustered based on the BP similarity. The size was positively correlated with the number of genes in this term. Each edge represents there were genes shared by these two BP terms, and the thickness was related to the number of shared genes.

**Abbreviation:** GO: gene ontology; BP: biological process.
